# VURD Syndrome in a Female

**DOI:** 10.1155/2011/852928

**Published:** 2011-01-05

**Authors:** A. Zaccara, M. P. Pascali, A. Marciano, E. Carnevale, G. Salvatori, A. Dotta, A. Nahom, M. De Gennaro

**Affiliations:** ^1^Department of Urology, Bambino Gesu' Children's Hospital, 00165 Rome, Italy; ^2^Department of Radiology, Bambino Gesu' Children's Hospital, 00165 Rome, Italy; ^3^Department of Medical and Surgical Neonatology, Bambino Gesu' Children's Hospital, 00165 Rome, Italy

## Abstract

VURD syndrome has been repeatedly described as unilateral reflux into a nonfunctioning renal moiety. This syndrome is considered a pop-off mechanism dissipating pressure in lower urinary tract obstruction: it may be found in association with other protective mechanisms occurring in utero, such as ascites and/or urinomas, and has been exclusively described in male patients. A premature female baby with signs and symptoms of outflow obstruction underwent diagnostic workup revealing congenital urethral hypoplasia with unilateral reflux into a dysplastic kidney. Obstetrical history was positive for early onset, serologically negative ascites without cardiomegaly, which required serial aspirations. Reconstructive surgery was carried out with good results: ascites and VURD syndrome were both deemed to be perinatal protective mechanism against excess pressure in the urinary tract. Although rare, lower urinary tract obstruction in the female can lead to the same protective mechanisms seen in male fetuses/newborns. VURD syndrome and ascites should be interpreted as such and require perinatal specialist counselling.

## 1. Introduction

Antenatal diagnosis of lower urinary tract obstruction has long been established: obstruction is usually associated with either posterior urethral valves or urethral atresia: as such, it is mostly seen in male fetuses [1]. Postnatal functional consequences of obstruction may consist of unilateral reflux and dysplasia, the so-called “VURD Syndrome” which is present in about 30% of patients with lower urinary tract obstruction. This syndrome was described in 1982 by Hoover and Duckett: a pop-off mechanism, dissipating high pressures generated by obstruction, was called upon to explain the association [2].

 Fetal lower urinary tract obstruction in the female is quite rare and mostly includes prolapsing ureterocele and cloacal plate anomalies: the former may obstruct bladder outlet [3] whereas in the latter failure of urogenital membrane to involute will result in accumulation of drainage above the membrane and may result in hydrocolpos, urethral or ureteral obstruction, and hydronephrosis [4]. 

Antenatally, a spectrum of protective mechanisms may be exerted by the fetus to relieve pressure within the urinary tract: ascites (secondary to microscopic perforation in the posterior fornix or with extravasations at the caliceal fornix with escaping urine reaching perirenal space [5]), bladder rupture [6], and more importantly, perinephric urinoma [7].

However, there are no reports in the literature of VURD syndrome occurring in female fetuses with evidence of protective mechanisms (ascites) occurring in utero.

## 2. Case Report

A premature, 30-week-gestation, female baby was transferred to our NICU with respiratory distress and ascites. Birth weight was 1120 gr, and she was the product of a twin dizygotic pregnancy. At 23-week-gestation the mother had a sonogram which revealed a female fetus with significant ascites: upon obstetrical advice, ascites required aspiration which was repeated five times because of rapid recurrence. No liver or spleen enlargement or cardiomegaly were present. The rest of ultrasound was unremarkable: no dilatations of urinary tract were described. Serology was negative. Elective C-section was performed at 30-week-gestation for maternal gestosis.

The baby required jet ventilation for 30 days; physical examination revealed a single anterior opening at perineum and patent anus but no visible urethral orifice. Karyotype was XX normal female.

**Figure 1:**
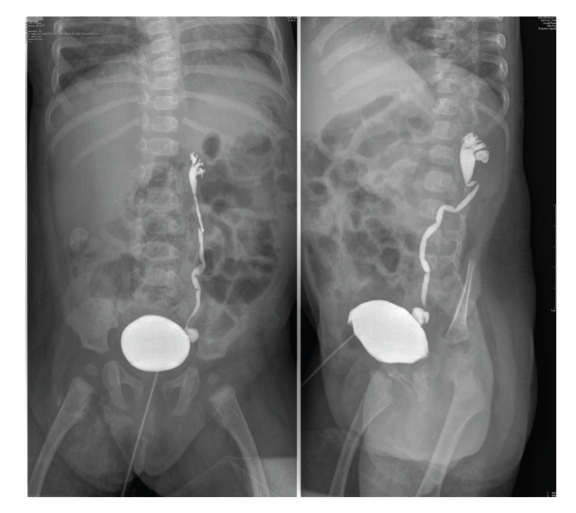
Voiding cystourethrogram showing unilateral reflux.

**Figure 2:**
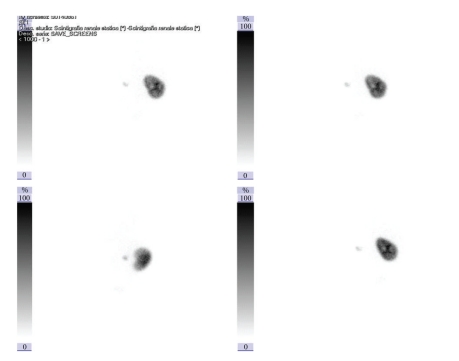
Nuclear scan demonstrating severely impaired function on the left side.

Abdominal US confirmed ascites with bladder distention: right kidney showed a mild pelvic dilatation whereas the left one was not visualized. Catheterization was impossible and a suprapubic tube was positioned. Drainage of urine led to prompt ascites resolution: the baby was weaned from ventilation after 30 days and S/P tube was clamped thereafter; this led to ascites recurrence so that vesicostomy was carried out. Renal function was always kept within normal limits. Further urological workup included a voiding cystourethrogram showing reflux into the left ureter (Figure 1) and a nuclear scan which revealed impaired function in the left kidney (Figure 2).

**Figure 3:**
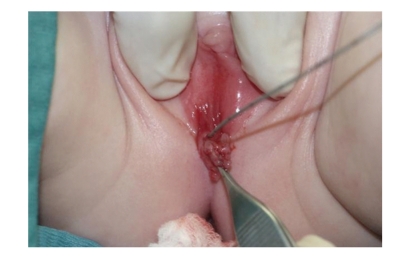
Intraoperative picture showing a 2F ureteral catheter exiting a tiny urethral meatus. A Hegar dilator is inserted in the vagina.

**Figure 4:**
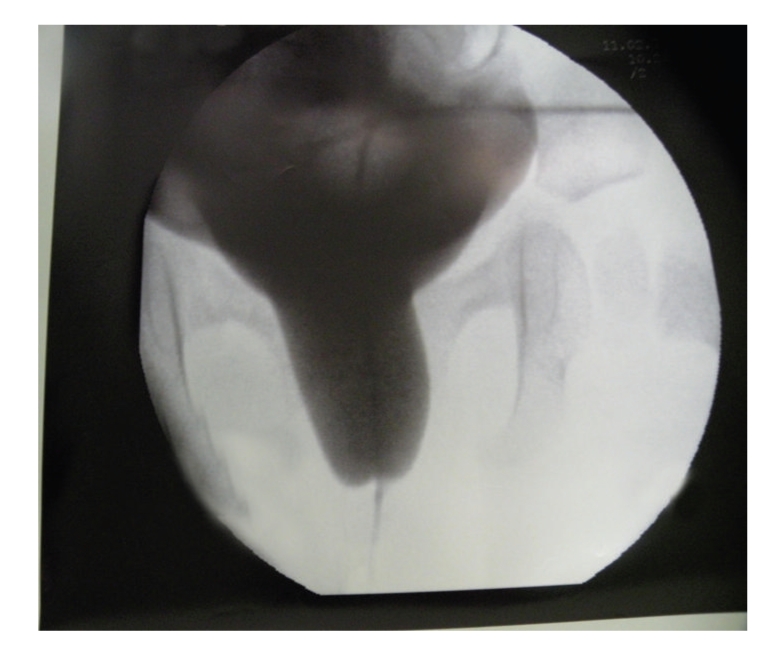
Intraoperative cystourethrogram showing sudden narrowing of the urethra.

At six months of age the baby underwent an exam under anesthesia which demonstrated a severely hypoplastic urethral meatus, negotiating only a 2F ureteral catheter with normal vagina (Figure 3). A contrast study demonstrated a severely hypoplastic urethra in its distal segment (Figure 4). Surgical reconstruction was performed by mobilization of the normal urethra to the perineum via an anterior, transpubic, sagittal approach. Total urethral length was approximately 3 cm, and the narrowed segment was 1.5 cm. At six-month followup the baby was passing urine per urethra without difficulties and without urinary tract infection.

## 3. Discussion

Our patient showed clear signs of outflow obstruction since birth. Diagnostic workup and intraoperative findings enabled us to demonstrate that such obstruction was due to sudden narrowing of the urethra about 1.5 cm above the perineum, in other words a sort of urethral hypoplasia.

Not surprisingly, congenital urethral obstruction has been predominantly described in boys, in the form of both atresia [8] and posterior urethral valves [9]. 

 In the female, congenital urethral stricture was described in association with female hypospadias in a 2-year-old girl: the urethra was opening into the superior aspect of the anterior vaginal wall [10]. 

 Differently from this observation, in our case, urethral meatus was tiny but at perineal level, without any communication with vagina.

Neonatal US scan showed distended bladder with pyelectasis, suggesting lower urinary tract obstruction; however, because of female sex such diagnosis was initially ruled out neither was it raised when early catheterization was impossible and we were able to alleviate obstruction only with a suprapubic tube. Voiding cystourethrogram and nuclear scan were performed only after weaning from jet ventilation and revealed unilateral reflux into a nonfunctioning moiety.

As previously mentioned, outflow obstruction, unilateral reflux and dysplastic kidney were referred to as “VURD Syndrome”. After initial observation, numerous other papers have described such association [11], as well as the beneficial effect of reflux into nonfunctioning unit over the contralateral moiety: however, despite that renal function is reported normal in all cases, scars have been described in the functioning kidney, making the “protective” effect questionable [12]. To our knowledge, there have been no reports in the literature about VURD syndrome in a female.

In our case the association reported in the literature was secondary to lower urinary tract obstruction induced by urethral hypoplasia: based on these findings, it seems reasonable to advocate the same pop-off mechanism dissipating high pressure seen in males.

We do not have an explanation for absence of bladder distention antenatally: however, the large amount of ascites, requiring up to five aspirations, may have impaired adequate visualization of the urinary tract; early (30 weeks ga) delivery also prevented further sonographic evaluation of the fetus later in pregnancy.

Interestingly, pregnancy was complicated by early onset, recurrent ascites. 

Fetal ascites without cardiomegaly or liver and spleen enlargement usually suggests urine ascites. Prenatal ascites has been repeatedly reported in female fetuses with cloaca: the hypothesis is that fetal urine drains through the uterine tubes into the peritoneal cavity [13]. The same association was reported for pure urogenital sinus [14]. Based on these findings, and given absence of visible urethral meatus on physical examination, it was not surprising that urogenital sinus (anus was normal) was the most likely diagnosis: it was not until surgical correction that we were able to detect a tiny urethral meatus, completely separated from vagina.

On the contrary, the cause of obstruction was a congenital urethral hypoplasia: therefore, it seems reasonable to speculate that even ascites was a sort of protective mechanism occurring in utero with urine backflow through the tubes into peritoneal cavity. In other words, excess pressure within the urinary tract might have dissipated partly in the urinary tract (leading to VURD association) and partly into the fetal/neonatal abdominal cavity (leading to ascites). In this respect, the therapeutic drainage procedures performed in utero (up to five) might have contributed in relieving excess pressure within the urinary tract thereby preserving renal function after birth. Direct correlation between ascites formation and obstruction could be supported by ascites recurrence after aspiration as well as after clamping of suprapubic tube as happened during perinatal management of our patient.

We believe that the reported case has unique features in that unilateral reflux into nonfunctioning kidney is characteristic of the VURD syndrome, exclusively seen in males; furthermore, recurrent ascites is likely to represent a fetal protective mechanism somewhat preserving renal function. 

Whatever the case is, it reinforces the assumption that serologically negative, isolated ascites without visceromegaly should suggest urine ascites; if this is the case, caution should be exerted in presence of rapid recurrence after aspiration, since it may represent a protective mechanism against obstruction, which may occur even in female fetuses.
